# The Mediating Role of Exercise in Depression and Fatigue in Patients with Advanced Cancer

**DOI:** 10.3390/curroncol31060229

**Published:** 2024-05-28

**Authors:** Caterina Calderon, Marina Gustems, Berta Obispo, Teresa García-García, Raquel Hernández, Ana Fernández-Montes, Paula Jiménez-Fonseca

**Affiliations:** 1 Faculty of Psychology, University of Barcelona, 08007 Barcelona, Spain; 2 Department of Medical Oncology, Hospital Infanta Leonor, 28031 Madrid, Spain; 3 Department of Medical Oncology, Hospital General Universitario Santa Lucia, 30202 Cartagena, Spain; 4 Department of Medical Oncology, Hospital Universitario de Canarias, 38320 Tenerife, Spain; 5 Department of Medical Oncology, Complejo Hospitalario Universitario de Ourense, 32005 Ourense, Spain; 6 Department of Medical Oncology, Hospital Universitario Central de Asturias, Instituto de Investigación del Principado de Asturias, ISPA, 33011 Oviedo, Spain

**Keywords:** exercise, fatigue, advanced cancer, psychological distress, quality of life

## Abstract

This study explored the interconnections between sociodemographic elements, depression, fatigue, and exercise in patients suffering from incurable neoplasm, particularly emphasizing the mediating influence of exercise on the relationship between depression and fatigue This was a prospective, multicenter, observational study involving 15 hospitals across Spain. After three months of systemic cancer treatment, participants completed the Brief Symptom Inventory (BSI), the Godin-Shephard Leisure-Time Physical Activity Questionnaire (GSLTPAQ) and the Fatigue Assessment Scale (FAS) to measure levels of depression, fatigue, and exercise, respectively. A total of 616 subjects participated in this study. Activity levels differed markedly according to educational attainment, marital, and work status. There was a negative correlation between physical activity and depression, and a positive correlation between depression and fatigue (β = −0.18, and β = 0.46, respectively). Additionally, physical activity inversely influenced fatigue levels (β = 0.21). Physical activity served as a partial intermediary in the link between depression and fatigue among patients with advanced, unresectable cancer. Healthcare providers are urged to consider both the physical and emotional dimensions of cancer treatment, implementing physical activity programs to enhance overall patient quality of life and mental health.

## 1. Introduction

Cancer and its treatment are commonly associated with lasting physical and psychosocial effects, leading to an increased risk of depression, fatigue, and diminished physical health and quality of life [1,2]. The psychological burden of a cancer diagnosis, uncertainties about prognosis and disease course, and the treatment side effects contribute to an increased depression risk in cancer patients [3,4]. Depression reduces treatment compliance, prolongs hospital stays, decreases quality of life, and hinders self-care capacity [5,6].

Fatigue is the most common side effect among cancer patients, with a prevalence ranging from 60% to 90% among those undergoing active treatment [7,8,9]. Characterized by deep, lasting physical, emotional, and cognitive exhaustion unrelated to recent exertion, it considerably interferes with daily activities and social connections, and may even predict survival outcomes [10,11]. Furthermore, it can persist for months or even years after treatment [3,7]. Moreover, the relationship between fatigue and depression in cancer patients is particularly concerning, as these conditions often co-occur and exacerbate each other’s effects. The emotional burden of cancer-related fatigue is significant, with the persistent lack of energy leading to feelings of helplessness and hopelessness, key symptoms of depression. This interplay between fatigue and depression can create a debilitating cycle, where fatigue worsens the symptoms of depression, and, in turn, depression increases the sensations of fatigue [10,11]. Research indicates that the emotional and cognitive components of fatigue among cancer patients may even predict survival outcomes. This suggests that fatigue is not just a side effect of cancer or its treatment, but a significant indicator of the disease’s progression. Furthermore, the persistence of fatigue, lasting for months or even years after the conclusion of treatment, poses a long-term challenge for survivors, impacting their quality of life and overall health outcomes [7,8,9]. Addressing both fatigue and depression is crucial in the clinical management of cancer patients. Interventions aimed at alleviating fatigue can significantly improve patients’ emotional well-being, potentially reducing the incidence or severity of depression. Likewise, treating depression in cancer patients may provide a dual benefit by also reducing perceived fatigue levels, thereby enhancing patients’ ability to engage in daily activities and maintain social connections, which are vital for emotional support and recovery [3,7].

In this context, physical exercise emerges as a critical element for cancer patients, as it can improve mood, reduce fatigue, and alleviate treatment side effects [1,12,13]. Additionally evidence suggests that exercise not only diminishes recurrence risk and boosts survival rates but also markedly improves quality of life [14]. Considered a potent non-pharmacological intervention, physical exercise promotes overall well-being in cancer patients [1]. Despite mixed findings regarding its impact on fatigue, the preponderance of systematic reviews affirms the positive effects of exercise on mood and fatigue levels [1,15,16,17,18,19]. Discrepancies between studies may be attributed to differences in research methodologies. In this context, physical exercise emerges as an important component for cancer patients, improving mood, reducing fatigue, and mitigating the side effects associated with cancer treatments. Additionally, science confirms that regular physical activity not only reduces the risk of cancer recurrence and increases survival rates but also significantly improves the quality of life for these patients. Considered an effective non-pharmacological intervention, physical exercise promotes overall well-being in individuals affected by cancer [1,15,16,17,18,19]. Although studies present varied results regarding the direct impact of exercise on fatigue, many systematic reviews confirm its beneficial effects on mood and fatigue levels.

The existing literature highlights a correlation between depression and fatigue, suggesting that physical exercise could act as a mechanism to cope with the stress derived from the disease and possibly mediate between depressive symptoms and fatigue [1,20]. Furthermore, exercise is considered a strategy to manage the stress stemming from cancer and its related challenges [1,20]. To date, no research has explored the mediating role of exercise in the interplay between depression and fatigue in patients with metastatic cancer in Spain. Therefore, this study aims to examine the connections between sociodemographic data, depression, exercise, and fatigue specifically focusing on the mediating role of exercise in these variables in patients with unresectable advanced cancer. The hypothesis posits that exercise will be a determining factor in the relationship between depression and fatigue.

## 2. Materials and Methods

### 2.1. Study Design and Population

**Figure 1:**
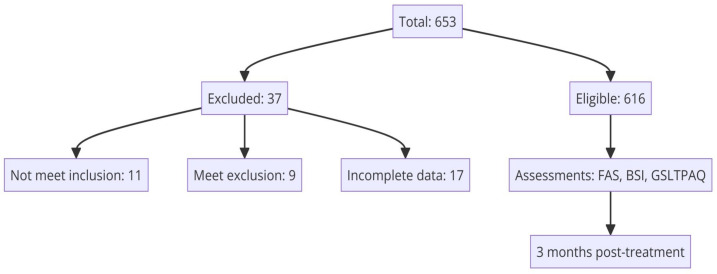
Flow chart depicting the inclusion and exclusion criteria of study participants.

Between February 2020 and November 2023, a cross-sectional study was implemented across 15 medical oncology units in various Spanish university hospitals (see Appendix A-Table A1). Eligible participants were adults aged 18 or older with a histopathologically verified diagnosis of unresectable advanced cancer who were not suitable for surgical or other curative interventions and were candidates for systemic cancer therapies. The exclusion criteria included individuals with any physical or mental conditions deemed by the oncologist as barriers to participation. Additionally, patients previously treated for another advanced cancer within the last two years or those facing significant medical, social, family, or personal challenges that could interfere with study involvement were also excluded. This also extended to individuals with cognitive disabilities, substantial overall health decline, or those unable to understand or respond to the study questionnaires. Recruitment occurred during the initial oncology consultation, where patients received detailed explanations about their diagnosis, the stage of their disease, and available systemic anticancer treatments. Those consenting to participate signed an informed consent form and were provided with questionnaires to fill out and return during their next visit. The study obtained approval from the Ethics Review Committee of each hospital involved and the Spanish Agency of Medicines and Health Products (AEMPS; identification code: ES14042015). Participation was voluntary, anonymous, and designed to ensure no interference with the patients’ standard care. Of the 653 individuals recruited for the study, 616 met the eligibility criteria. Thirty-seven participants were excluded for various reasons: 11 did not meet the inclusion criteria, 9 met at least one exclusion criterion, and 17 provided incomplete data. Figure 1 provides a flow chart that details the inclusion and exclusion criteria for participants in the study. 

### 2.2. Description of Variables

Data collection methods for the study included administering questionnaires, conducting interviews, and examining medical records, which were uniformly implemented across all involved hospitals. Medical oncologists managed the data using the web-based platform (www.neoetic.es, accessed date on 1 November 2023). Information on sociodemographic factors was collected through a standardized self-report form, while medical histories provided disease-related data. Three months post-initiation of systemic treatment, patients were required to complete the Fatigue Assessment Scale (FAS), the Brief Symptom Inventory (BSI), and the Godin-Shephard Leisure-Time Physical Activity Questionnaire (GSLTPAQ), aligning this assessment with the timing of the CT scans used to evaluate antineoplastic treatment responses.

Fatigue levels were quantified using the FAS [21], which includes 10 items—five targeting physical fatigue and five targeting mental fatigue. Responses are rated on a 5-point scale, ranging from 1 (“never”) to 5 (“always”), with total scores varying from 10 to 50; higher scores signify greater fatigue severity. The scale is noted for its high internal consistency, evidenced by a Cronbach’s alpha of 0.90 [22]. The Spanish version of the FAS has been validated for reliability and accuracy [23].

Depression was evaluated using the depression subscale of the BSI [24], which encompasses six descriptions that capture both the physical and emotional aspects of depression, including feelings of worthlessness, lack of pleasure, despair, and thoughts of self-harm. Scoring for this subscale is based on a 5-point Likert scale. Higher scores indicate more severe symptoms of depression. Scores are then transformed to T-scores, reflecting gender-specific norms. The BSI identifies potential depression in individuals who exceed a T-score threshold of 63, as per the clinical case-rule suggested by Derogatis [24], indicating ‘probable depression’. The Spanish version of this tool also demonstrates commendable reliability and validity [25].

Exercise levels were evaluated using the Godin-Shephard Leisure-Time Physical Activity Questionnaire (GSLTPAQ) [26], which measures the intensity of physical activity (ranging from none to vigorous) and records the frequency (from less than once a month to 3–5 times a week) and duration (from less than 10 min to more than 30 min) of these activities. This scale has been utilized in research with patients suffering from breast, prostate, and colorectal cancer [27].

### 2.3. Statistical Methods

Statistical analysis was performed using SPSS for Windows 26.0 (SPSS Inc., Chicago, IL, USA). All tests were two-sided, and significance was set at *p* <  0.05. Descriptive statistics for demographic variables included the mean, standard deviation (SD), counts (N), and percentage (%). Differences in depression, exercise, and fatigue across groups were assessed using *t*-tests and one-way ANOVA. Effect sizes were denoted by eta-squared (η^2^), ranging from 0 to 1, with η^2^ ~0.01 indicating a small, η^2^ ~0.06 a medium, and η^2^ > 0.14 a large effect size [28]. Pearson’s correlation was used to examine relationships between continuous variables. Multiple hierarchical regression analyses were conducted to explore the relationship between exercise, depression, and fatigue using MedGraph I software 3.0 version for mediation analysis. Following Baron and Kenny’s mediation conditions [29], it is required to demonstrate the following: (1) a significant relationship between depression and fatigue, indicating that depressive states may be associated with levels of fatigue; (2) a significant relationship between depression and exercise, suggesting that depression can influence the amount or intensity of exercise performed; and (3) a significant relationship between exercise and fatigue, demonstrating that exercise can act as a mediator in the relationship between depression and fatigue. The mediation effect size was calculated using Sobel’s test.

## 3. Results

### 3.1. Sociodemographic and Clinical Characteristics 

Detailed characteristics of the study population are presented in Table 1 and Table 2. The cohort comprised 53.4% males (*n* = 329) and 46.6% (*n* = 287) female. The mean age was around 65.0 years (standard deviation = 10.2). Bronchopulmonary (32.0%), colorectal (16.2%), breast (11.0%), and pancreatic (9.7%) cancers were the most common types observed, with adenocarcinoma as the predominant histology (69.6%). Of these, 80.8% were metastatic, and 19.2% were locally advanced and unresectable. The primary treatments included chemotherapy (47.7%), targeted therapy (7.0%), and immunotherapy (6.8%) or combinations of these (38.5%). Approximately 42.9% of the participants were estimated to have a survival time of less than 12 months.

Significant gender differences were observed in depression levels, with higher depression scores in women than in men (*F* = 35.486, *p* = 0.001, η^2^ = 0.055), whereas no significant differences were found in exercise (*p* = 0.204) or fatigue (*p* = 0.098). Age was significantly associated with variations in depression (*F* = 6.009, *p* = 0.003, η^2^ = 0.019) and exercise (*F* = 5.194, *p* = 0.006, η^2^ = 0.017), but not with fatigue (*p* = 0.774). Individuals ≤60 years of age exhibited the highest levels of depression, while exercise was lower in individuals over 70 years old. Marital status differences impacted depression significantly (*F* = 4.535, *p* = 0.034, η^2^ = 0.007), with married or partnered individuals showing lower depression scores. Educational level had a significant impact on exercise (*F* = 42.783, *p* = 0.001, η^2^ = 0.065), with individuals who had completed high school or attained a higher level of education achieving higher scores. Employment status had a significant impact on exercise (*F* = 4.703, *p* = 0.031, η^2^ = 0.008) and fatigue (*F* = 5.974, *p* = 0.015, η^2^ = 0.010); employed individuals showed higher levels of both exercise and fatigue. 

In examining the relationships between depression, exercise, fatigue, and patient characteristics within the study group (Table 2), no statistically significant differences were observed based on tumor location, histological type, or treatment modality. However, significant differences were identified in relation to the performance status as assessed by the Eastern Cooperative Oncology Group (ECOG) scale. Patients with an ECOG of 1 or more, who exhibit greater functional limitations, showed higher levels of depression (mean ± SD: 62.1 ± 6.5) and fatigue (mean ± SD: 25.0 ± 6.9), along with a substantial reduction in their exercise levels (mean ± SD: 46.4 ± 29.6) compared to those with an ECOG of 0 (depression: mean ± SD: 59.9 ± 6.1; exercise: mean ± SD: 65.4 ± 26.3; fatigue: mean ± SD: 23.3 ± 6.3). These findings underscore how deterioration in functional capacity can negatively impact patients’ physical and emotional well-being, significantly affecting their quality of life.

### 3.2. Correlations across Variables

Table 3 displays the means, standard deviations, and Pearson correlation analyses for the studied variables. Mean scores for depression, exercise, and fatigue were 61.4 ± 6.4, 54.9 ± 29.7, and 24.2 ± 6.7, respectively. Analysis revealed significant correlations across all psychological variables, aligning with the hypotheses: depression was inversely related to exercise and directly related to fatigue, whereas exercise inversely affected fatigue levels. These findings satisfy the initial two conditions of Baron and Kenny’s mediation analysis technique as applied in this study.

### 3.3. The Mediating Role of Exercise in the Relationship between Depression and Fatigue

**Figure 2:**
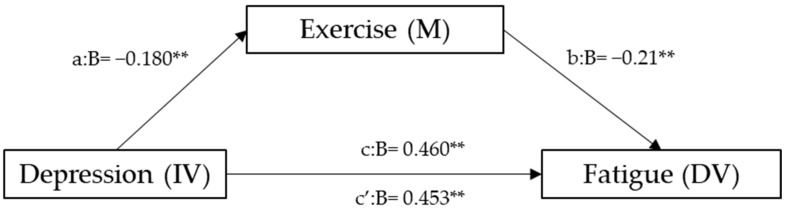
Mediation model for depression, exercise, and fatigue. a = direct effect of independent variable (IV) on mediator (M). b = direct effect of mediator on dependent variable (DV). c = direct effect of IV on DV. c’ = indirect effect of IV on DV. ** *p* < 0.01.

Hierarchical linear regression analyses to explore the mediating role of exercise are represented in Figure 2. After adjusting for sociodemographic factors, the results reveal that depression was negatively associated with exercise and positively with fatigue (*β* = −0.18, and *β* = 0.46, respectively), whereas exercise correlated negatively with fatigue (*β* = 0.21). The mediation analysis, assessed through the Sobel test, yielded a highly significant z-value of 298.406 (*p* = 0.002845), confirming the statistical significance of the mediation effect. The 95% confidence interval for the unstandardized indirect effect (a*b) ranged from 0.00911 to 0.04396, emphasizing the robustness of the mediation. Effect size measures indicated a moderate-sized total effect (0.460), with a substantial direct effect (0.453). Notably, the indirect effect contributed significantly (0.025) to the overall effect, accounting for 99.3%. 

## 4. Discussion

This study is, to our knowledge, the first to explore the role of exercise in Spanish individuals with unresectable advanced cancer, particularly its mediating role between depression and fatigue in this specific population. The findings indicate that exercise acts as a mediator, accounting for 22.8% of the relationship, confirming the anticipated correlations among depression, exercise, and fatigue.

Our cohort of 616 participants presents an equitable gender distribution, with a slight predominance of men (53%). Depression emerges as a frequent symptom in cancer patients, affecting approximately one in four [30], with a higher prevalence in women and younger patients [31,32]. These findings, which show a greater occurrence of depressive symptoms in women and younger individuals, align with previous research [30,32]. Additionally, marital status emerged as a significant determinant of depression levels, underscoring the potential role of social support in mitigating depressive symptoms among married or partnered patients [33]. These results highlight the importance of addressing mental health interventions, particularly for certain subgroups, within the oncological population.

In our sample, younger patients and those with higher education levels engaged in more physical activity compared to their older or less educated counterparts. Variations in exercise levels among patients of different ages in the oncological context may be attributed to factors such as physical limitations associated with aging, comorbidities, and differences in attitudes and preferences towards exercise [1,34]. Younger individuals may show greater motivation and perception of exercise-related benefits, in addition to receiving more emphasized medical recommendations about its importance [1,35]. Likewise, the impact of education on physical activity was notable, highlighting the positive relationship between higher educational levels and increased participation in physical activity, consistent with the existing literature [36,37]. Furthermore, employment status significantly affected exercise frequency and fatigue, with employed individuals reporting more physical activity but also higher fatigue levels. These findings may suggest a complex interplay between occupation, physical activity, and well-being, possibly influenced by specific work factors such as workload or type of occupation.

Our series revealed an inverse association between depression and physical exercise, alongside a direct relationship between depression and fatigue. Depressive symptoms are common in cancer patients, with their prevalence estimated to be between 13% and 40% [2,7]. Similarly, fatigue is a common complaint, reported by 40% to 100% of patients during oncological treatments and by 14% to 40% following treatment completion [38,39]. Previous studies have documented the interconnectedness of depression and fatigue in cancer patients [2,39,40]. In particular, the emotional aspects and inner tension of fatigued patients exhibit similarities with anhedonia and psychomotor agitation, characteristic of depressive disorders [7,41]. The mediation analysis indicates that exercise plays a crucial role in mediating the relationship between depression and fatigue. This underscores the need to integrate physical activity interventions into the treatment plans for managing fatigue and its emotional consequences in depressed cancer patients.

The limitations of this study should be considered when considering its application. Firstly, the assessment is restricted to a single point in time, which precludes the establishment of causality. Secondly, while self-administered questionnaires at home are intended to give patients ample time to respond thoroughly and calmly, they may introduce biases due to misunderstandings. Thirdly, variations in cancer types and antineoplastic treatment modalities were not considered as potential modifiers of the outcomes. Lastly, the study involves a Spanish population, which has its own ethnic, cultural, and healthcare-specific characteristics. Additionally, the authors need to acknowledge potential biases, especially that patients with poor performance status due to disease progression or metastatic disease are more likely to be less active, as well as feel fatigued and depressed. They should also discuss how they might overcome or account for these factors.

## 5. Conclusions

This study provides preliminary insights into the care of people with advanced cancer, highlighting exercise as a potential mediator between depression and fatigue. It underscores the importance of integrating physical activity interventions into the clinical management of these patients and the necessity to develop exercise programs tailored to the specific requirements of this population to address the symptoms of depression and fatigue that they often experience. Furthermore, the observed variations in physical activity levels based on age, education, and employment status highlight the need for a personalized approach in recommending exercise, ensuring that interventions are as effective as possible. Ultimately, these findings highlight the necessity of a holistic care strategy that encompasses both physical and emotional health, positioning exercise as a key element in improving the quality of life and mental well-being of cancer patients.

## Figures and Tables

**Table 1:** Comparison of mean total scores for depression, exercise, and fatigue according to baseline sample characteristics.

Characteristics	N (%)	Depression (Mean ± SD)	Exercise (Mean ± SD)	Fatigue (Mean ± SD)
Sex				
Male	329 (53.4)	59.7 ± 5.6	56.3 ± 29.4	23.8 ± 6.7
Female	287 (46.6)	62.7 ± 6.9	53.3 ± 30.1	24.7 ± 6.6
*p* value		0.001	0.204	0.098
Age		35.486	1.617	2.753
≤60 years	186 (30.2)	62.4 ± 6.6	57.7 ± 30.6	24.2 ± 6.3
61–70 years	217 (35.2)	61.0 ± 5.9	57.8 ± 27.6	24.4 ± 6.9
>70 years	213 (34.6)	60.1 ± 6.5	49.6 ± 30.4	24.0 ± 6.8
*p* value		0.003	0.006	0.774
Marital Status		6.009	5.194	0.257
Married/partnered	419 (68.0)	60.7 ± 6.1	56.1 ± 29.2	24.4 ± 6.4
Not partnered	197 (32.0)	61.9 ± 6.9	52.5 ± 30.6	23.9 ± 7.2
*p* value		0.034	0.175	0.395
Educational level		4.535	1.848	0.724
Primary	272 (44.2)	61.1 ± 6.6	46.4 ± 29.8	24.4 ± 7.7
High school or higher	344 (55.8)	61.2 ± 6.2	61.6 ± 27.9	24.0 ± 5.8
*p* value		0.718	0.001	0.442
Employment		0.130	42.783	0.591
Unemployed	444 (72.1)	61.2 ± 6.4	53.3 ± 29.9	23.8 ± 6.5
Employed	172 (27.9)	61.1 ± 6.5	59.1 ± 28.8	25.3 ± 6.9
*p* value		0.822	0.031	0.015

Abbreviations: N, number; SD, standard deviation.

**Table 2:** Differences in clinical characteristics associated with depression, exercise, and fatigue among patients (*n* = 616).

Characteristics	N (%)	Depression (Mean ± SD)	Exercise (Mean ± SD)	Fatigue (Mean ± SD)
Tumor site				
Bronchopulmonary	197 (32.0)	61.1 ± 6.5	53.9 ± 28.2	23.9 ± 6.5
Colorectum	100 (16.2)	60.9 ± 6.5	54.8 ± 30.0	24.5 ± 6.9
Breast	68 (11.0)	60.4 ± 5.8	55.2 ± 29.2	23.4 ± 5.7
Pancreas	60 (9.7)	61.4 ± 6.4	51.6 ± 30.3	23.7 ± 6.8
Stomach	30 (4.9)	61.4 ± 7.3	55.1 ± 33.9	24.9 ± 7.1
Others	161 (26.1)	61.5 ± 6.4	57.2 ± 29.5	24.2 ± 6.7
*p* value		0.884	0.856	0.640
Histology				
Adenocarcinoma	429 (69.6)	60.9 ± 6.3	56.5 ± 29.2	24.4 ± 6.8
Others	187 (30.4)	61.7 ± 6.6	51.3 ± 30.7	24.5 ± 6.4
*p* value		0.052	0.138	0.455
Stage				
Locally Advanced	118 (19.2)	61.2 ± 6.9	50.0 ± 31.7	25.3 ± 7.1
Metastatic (IV)	498 (80.8)	61.1 ± 6.3	52.5 ± 30.6	24.0 ± 6.5
*p* value		0.870	0.058	0.059
Type of treatment				
Only CT	294 (47.7)	61.2 ± 6.5	50.4 ± 30.7	24.9 ± 7.1
CT with other	237 (38.5)	61.0 ± 6.2	58.4 ± 28.1	23.6 ± 6.1
Immunotherapy	43 (7.0)	60.6 ± 6.4	56.8 ± 31.8	23.6 ± 7.0
Targeted therapies	42 (6.8)	62.1 ± 7.0	58.0 ± 25.4	23.8 ± 6.7
*p* value		0.386	0.143	0.081
ECOG-PS				
0	274 (44.5)	59.9 ± 6.1	65.4 ± 26.3	23.3 ± 6.3
1 or more	342 (55.5)	62.1 ± 6.5	46.4 ± 29.6	25.0 ± 6.9
*p* value		0.001	0.001	0.002

Abbreviations: CT, chemotherapy; ECOG-PS, Eastern Cooperative Oncology Group performance status; N, number; SD, standard deviation.

**Table 3:** Correlations between depression, exercise, and fatigue scores.

	Mean	SD	Depression	Exercise
Depression	61.2	6.4	1	
Exercise	54.9	29.7	−0.184 **	1
Fatigue	24.2	6.7	0.460 **	−0.219 **

Abbreviations: SD, standard deviation: ** *p* < 0.001.

## Data Availability

The datasets generated and analyzed during the current study are not publicly available for reasons of privacy. They are, however, available (fully anonymized) from the corresponding author on reasonable request.

## Appendix A

**Table A1:** List of 15 centers participating in the study.

(1)	Hospital Universitario de Canarias, Tenerife
(2)	Hospital Universitario La Paz, Madrid
(3)	Hospital Universitario Central de Asturias, Oviedo
(4)	Complejo Hospitalario Universitario de Ourense
(5)	Hospital Universitario Infanta Leonor, Madrid
(6)	Consorcio Hospital General Universitario de Valencia, Valencia
(7)	Hospital General Virgen de la Luz, Cuenca
(8)	Hospital Provincial de Castellón, Castellón
(9)	Hospital General Universitario de Elche, Elche
(10)	Hospital Universitario Clínico San Carlos, Madrid
(11)	Hospital San Pedro de Alcántara, Cáceres
(12)	Hospital Universitario Morales Meseguer, Murcia
(13)	Hospital Quironsalud Sagrado Corazón de Sevilla, Sevilla
(14)	Hospital General Universitario Santa Lucia, Cartagena
(15)	Hospital General de Ciudad Real, Ciudad Real
